# Quantitative unique continuation for the linear coupled heat equations

**DOI:** 10.1186/s13660-017-1508-7

**Published:** 2017-09-21

**Authors:** Guojie Zheng, Keqiang Li, Jun Li

**Affiliations:** College of Mathematics and Information Science, Henan Normal University, Xinxiang, 453007 P.R. China

**Keywords:** 35K05, 93D15, coupled heat equations, unique continuation, frequency functions

## Abstract

In this paper, we established a quantitative unique continuation results for a coupled heat equations, with the homogeneous Dirichlet boundary condition, on a bounded convex domain Ω of $\mathbb{R}^{d}$ with smooth boundary *∂*Ω. Our result shows that the value of the solutions can be determined uniquely by its value on an arbitrary open subset *ω* of Ω at any given positive time *T*.

## Introduction

In this paper, we consider an unique continuation of the following linear coupled heat equations:

1.1 $$ \textstyle\begin{cases} y_{t}(x,t)-\triangle y(x,t)-a(x,t)y(x,t)-b(x,t)z(x,t)=0, & (x,t) \in\Omega\times(0,T), \\ z_{t}(x,t)-\triangle z(x,t)-c(x,t)y(x,t)-d(x,t)z(x,t)=0, & (x,t) \in\Omega\times(0,T), \\ y(x,t)=0, & (x,t)\in\partial\Omega\times(0,T), \\ z(x,t)=0, & (x,t)\in\partial\Omega\times(0,T), \end{cases} $$

where Ω is a bounded, convex domain of $\mathbb{R}^{d}$ ($d \geq1$), with a smooth boundary *∂*Ω. Let $\omega \subset\Omega$ be a nonempty and open subset of Ω, and *T* is a positive number. Now we suppose that

1.2 $$\begin{aligned} a(x,t),b(x,t),c(x,t),d(x,t)\in L^{\infty}\bigl(\Omega \times(0,T)\bigr) \end{aligned}$$

and

1.3 $$\begin{aligned} \max\bigl\{ \Vert a\Vert _{L^{\infty}(\Omega\times(0,T))},\Vert b\Vert _{L^{\infty}( \Omega\times(0,T))},\Vert c\Vert _{L^{\infty}(\Omega \times(0,T))},\Vert d\Vert _{L^{\infty}(\Omega\times(0,T))} \bigr\} \leq M, \end{aligned}$$

where *M* is a positive number.

In this paper, we are concerned with the unique continuation for the solution of Eq. (1.1). The main results obtained in this work can be stated as follows.

### Theorem 1.1

*Let* *ω* *be a nonempty open subset of* Ω. *Then there are two positive numbers* $p=p(\Omega,\omega)$ *and* $C=C(\Omega,\omega)$ *such that for each* $T>0$ *and for each potential* $a, b, c, d\in L^{\infty}( \Omega\times(0,T))$ *with condition* (1.3), *any solution* $(y,z)$ *with* $y(\cdot,0)\equiv y_{0}(\cdot)\in L^{2}(\Omega)$, $z(\cdot,0)\equiv z_{0}(\cdot)\in L^{2}(\Omega)$, *to equation* (1.1) *has the following estimate*:

1.4 $$\begin{aligned}& \int_{\Omega}\bigl(\bigl\vert y(x,T)\bigr\vert ^{2}+ \bigl\vert z(x,T)\bigr\vert ^{2}\bigr)\,dx \\& \quad \leq C\exp \biggl( \frac{C}{T}+C\bigl(MT+M ^{2}T^{2}\bigr) \biggr) \times \biggl( \int_{\Omega}\bigl(\bigl\vert y_{0}(x)\bigr\vert ^{2}+\bigl\vert z_{0}(x)\bigr\vert ^{2} \bigr)\,dx \biggr) ^{1-p} \\& \quad\quad {}\times \biggl( \int_{\omega}\bigl(\bigl\vert y(x,T)\bigr\vert ^{2}+ \bigl\vert z(x,T)\bigr\vert ^{2}\bigr)\,dx \biggr) ^{p}. \end{aligned}$$

### Theorem 1.2

*Let* *ω* *be a nonempty open subset of* Ω. *If the initial data* $(y_{0}, z_{0})\not\equiv(0,0)$, *and* $y_{0}, z_{0}\in H_{0} ^{1}(\Omega)$, *then there exists a positive number* $C=C(\Omega, \omega)$ *such that for each* $T>0$ *and for each potential* $a, b, c, d \in L^{\infty}(\Omega\times(0,T))$ *with condition* (1.3), *any solution* $(y,z)$ *with* $y(\cdot,0)\equiv y_{0}(\cdot)$, $z(\cdot,0)\equiv z_{0}(\cdot)$, *to equation* (1.1) *has the following estimate*:

1.5 $$\begin{aligned}& \Vert y_{0}\Vert ^{2}_{L^{2}(\Omega)}+ \Vert z_{0}\Vert ^{2}_{L^{2}(\Omega)} \\& \quad \leq Ce ^{\frac{C}{T}}e^{C(MT+M^{2}T^{2})}\exp \biggl( C(1+2M)Te^{CM^{2}T} \frac{ \Vert y_{0}\Vert ^{2}_{H_{0}^{1}(\Omega)}+\Vert z_{0}\Vert ^{2}_{H_{0}^{1}(\Omega)}}{ \Vert y_{0}\Vert ^{2}_{L^{2}(\Omega)}+\Vert z_{0}\Vert ^{2}_{L^{2}(\Omega)}} \biggr) \\& \quad\quad{} \times \int_{\omega}\bigl(\bigl\vert y(x,T)\bigr\vert ^{2}+ \bigl\vert z(x,T)\bigr\vert ^{2}\bigr)\,dx. \end{aligned}$$

### Remark 1.1

The constant *C* in (1.4) or (1.5) stands for a positive constant only dependent on Ω, *ω*. This constant varies in different contexts.

The study of unique continuation for the solutions for PDEs began at the early of the last centaury. Besides its own interesting in PDEs theory, it also plays a key role in both inverse problem and control theory. The first quantitative result of strong unique continuation for parabolic equations was derived in 1974 in the literature [1]. In [1], the authors establish the unique continuation for the parabolic equations with time independent coefficients in terms of the eigenfunctions of the corresponding elliptic operator, and their results did not apply to parabolic equations with time dependent coefficients. After 1988, there were more results of unique continuation for parabolic equations, and we refer the reader to [2–9], and the rich work cited therein. In [10], the author discusses the unique continuation for stochastic counterpart. In our paper, we mainly study this property for the coupled heat equations. To the best of our knowledge, this topic has not been studied in past publications. With the aid of frequency function methods, we can establish these quantitative estimates (see [2, 6]).

The paper is organized as follows. In Section 2, some preliminary results are presented. The proofs of Theorem 1.1 and Theorem 1.2 will be given in Section 3.

## Preliminary results

Given a positive number *λ*, we define

2.1 $$\begin{aligned} G_{\lambda}(x,t)=\frac{1}{(T-t+\lambda)^{d/2}}e^{-\frac{\vert x-x_{0}\vert ^{2}}{4(T-t+ \lambda)}},\quad (x,t)\in\Omega\times(0,T). \end{aligned}$$

Then, for each $t\in[0,T]$, we write

2.2 $$\begin{aligned}& H_{\lambda}(t)= \int_{\Omega}\bigl(\bigl\vert y(x,t)\bigr\vert ^{2}+ \bigl\vert z(x,t)\bigr\vert ^{2}\bigr)G_{\lambda}(x,t)\,dx, \end{aligned}$$

2.3 $$\begin{aligned}& D_{\lambda}(t)= \int_{\Omega}\bigl(\bigl\vert \nabla y(x,t)\bigr\vert ^{2}+\bigl\vert \nabla z(x,t)\bigr\vert ^{2}\bigr)G _{\lambda}(x,t)\,dx, \end{aligned}$$

and

2.4 $$\begin{aligned} N_{\lambda}(t)=\frac{2D_{\lambda}(t)}{H_{\lambda}(t)}, \end{aligned}$$

where $(y(x,t), z(x,t))$ are the solutions of equation (1.1). The function $N_{\lambda}(t)$ was first discussed in [11], and it was called frequency function (see also [2, 12], and [9]). Throughout this section, we always work under the assumption $H_{\lambda}(t)\neq0$. Next, we will discuss the properties for the functions $G_{\lambda}(x,t)$, $H_{\lambda}(t)$, $D_{\lambda}(t)$ and $N_{\lambda}(t)$. Now, we first fix a positive number *r* and a point $x_{0}$ in the subset *ω* such that $B_{r}\subset\omega$. $B_{r}$ denotes the open ball, centered at the point $x_{0}$ and of radius *r*, in $\mathbb{R}^{d}$. Write $m=\sup_{x\in\Omega} \vert x-x_{0}\vert ^{2}$. Lemma 2.1 is taken from [2, 6].

### Lemma 2.1

*For each* $\lambda>0$, *the function* $G_{\lambda}$ *given in* (2.1) *holds the following four identities over* $\mathbb{R}^{d}\times[0,T]$:

2.5 $$\begin{aligned}& \partial_{t}G_{\lambda}(x,t)+\triangle G_{\lambda}(x,t)=0, \end{aligned}$$

2.6 $$\begin{aligned}& \nabla G_{\lambda}(x,t)=\frac{-(x-x_{0})}{2(T-t+\lambda)}G_{\lambda }(x,t), \end{aligned}$$

2.7 $$\begin{aligned}& \partial_{i}^{2}G_{\lambda}(x,t)= \frac{-1}{2(T-t+\lambda)}G_{\lambda }(x,t)+\frac{\vert x_{i}-x_{0i}\vert ^{2}}{4(T-t+\lambda )^{2}}G_{\lambda}(x,t), \end{aligned}$$

*and*, *for* $i\neq j$,

2.8 $$\begin{aligned} \partial_{i}\partial_{j}G_{\lambda}(x,t)= \frac{(x_{i}-x_{0i})(x_{j}-x _{0j})}{4(T-t+\lambda)^{2}}G_{\lambda}(x,t). \end{aligned}$$

### Lemma 2.2

*For each* $\lambda>0$, *the following identity holds for* $t\in(0,T)$:

2.9 $$\begin{aligned} \frac{d}{dt}\ln{H_{\lambda}(t)}=-N_{\lambda}(t)+ \frac{2}{H_{\lambda }(t)} \int_{\Omega} \bigl( y(\partial_{t}y-\triangle y)+z( \partial_{t}z- \triangle z) \bigr) G_{\lambda}\,dx. \end{aligned}$$

### Proof

By a direct computation, we can obtain

2.10 $$\begin{aligned} H'_{\lambda}(t) =&2 \int_{\Omega}(y\partial_{t}y+z\partial_{t}z)G _{\lambda}\,dx+ \int_{\Omega}\bigl(y^{2}+z^{2}\bigr) \partial_{t}G_{\lambda}\,dx \\ =&2 \int_{\Omega}(y\partial_{t}y+z\partial_{t}z)G_{\lambda}\,dx- \int_{\Omega}\bigl(y^{2}+z^{2}\bigr)\Delta G_{\lambda}\,dx \\ =&2 \int_{\Omega}(y\partial_{t}y+z\partial_{t}z)G_{\lambda}\,dx- \int_{\Omega}\Delta\bigl(y^{2}+z^{2} \bigr)G_{\lambda}\,dx \\ =&2 \int_{\Omega}(y\partial_{t}y+z\partial_{t}z)G_{\lambda}\,dx-2 \int_{\Omega} \bigl( (\nabla y)^{2}+y\triangle y+(\nabla z)^{2}+z \triangle z \bigr) G_{\lambda}\,dx \\ =&2 \int_{\Omega} \bigl( y(\partial_{t}y-\triangle y)+z( \partial_{t}z- \triangle z) \bigr) G_{\lambda}\,dx-2 \int_{\Omega} \bigl( \vert \nabla y\vert ^{2}+\vert \nabla z\vert ^{2} \bigr) G_{\lambda}\,dx \\ =&2 \int_{\Omega} \bigl( y(\partial_{t}y-\triangle y)+z( \partial_{t}z- \triangle z) \bigr) G_{\lambda}\,dx-2D_{\lambda}(t). \end{aligned}$$

Therefore, for each $t\in(0,T)$, we have

2.11 $$\begin{aligned} \frac{d}{dt}\ln{H_{\lambda}(t)}=\frac{H'_{\lambda}(t)}{H_{\lambda }(t)}=-N_{\lambda}(t)+ \frac{2}{H_{\lambda}(t)} \int_{\Omega} \bigl( y( \partial_{t}y-\triangle y)+z( \partial_{t}z-\triangle z) \bigr) G_{ \lambda}\,dx. \end{aligned}$$

This completes the proof of this lemma. □

### Lemma 2.3

*For each* $\lambda>0$ *and* $t\in(0,T)$, *the functions* $H_{\lambda}$ *and* $D_{\lambda}$ *defined in* (2.2) *and* (2.3), *respectively*, *satisfy*

2.12 $$\begin{aligned} 2H'_{\lambda}(t)D_{\lambda}(t) =&- \biggl[2 \int_{\Omega}y \biggl( \partial_{t}y-\frac{x-x _{0}}{2(T-t+\lambda)} \nabla y+\frac{1}{2}(\triangle y-\partial _{t}y) \biggr) G _{\lambda}\,dx \\ &{}+2 \int_{\Omega}z \biggl( \partial_{t}z- \frac{x-x_{0}}{2(T-t+\lambda)} \nabla z+\frac{1}{2}(\triangle z- \partial_{t}z) \biggr) G_{\lambda}\,dx \biggr]^{2} \\ &{}+ \biggl( \int_{\Omega}y(\triangle y-\partial_{t}y)G_{\lambda}\,dx+ \int_{\Omega}z(\triangle z-\partial_{t}z)G_{\lambda}\,dx \biggr) ^{2}. \end{aligned}$$

### Proof

By (2.5), (2.6), we can rewrite $H'_{\lambda}(t)$ as follows:

2.13 $$\begin{aligned} H'_{\lambda}(t) =&2 \int_{\Omega}(y\partial_{t}y+z\partial_{t}z)G _{\lambda}\,dx- \int_{\Omega}\bigl(y^{2}+z^{2}\bigr)\triangle G_{\lambda}\,dx \\ =&2 \int_{\Omega}(y\partial_{t}y+z\partial_{t}z)G_{\lambda}\,dx+ \int_{\Omega}\nabla\bigl(y^{2}+z^{2}\bigr) \nabla G_{\lambda}\,dx \\ =&2 \int_{\Omega}(y\partial_{t}y+z\partial_{t}z)G_{\lambda}\,dx- \int_{\Omega}(2y\nabla y+2z\nabla z)\frac{x-x_{0}}{2(T-t+\lambda)}G _{\lambda}\,dx \\ =&2 \int_{\Omega}y \biggl( \partial_{t}y- \frac{x-x_{0}}{2(T-t+\lambda)} \nabla y \biggr) G_{\lambda}\,dx+2 \int_{\Omega}z \biggl( \partial_{t}z-\frac{x-x_{0}}{2(T-t+\lambda)} \nabla z \biggr) G_{\lambda}\,dx \\ =&2 \int_{\Omega}y \biggl( \partial_{t}y- \frac{x-x_{0}}{2(T-t+\lambda)} \nabla y+\frac{1}{2}(\triangle y- \partial_{t}y) \biggr) G_{\lambda}\,dx \\ &{}+2 \int_{\Omega}z \biggl( \partial_{t}z- \frac{x-x_{0}}{2(T-t+\lambda)} \nabla z+\frac{1}{2}(\triangle z- \partial_{t}z) \biggr) G_{\lambda}\,dx \\ &{}- \int_{\Omega}y(\Delta y-\partial_{t}y)G_{\lambda}\,dx- \int_{\Omega }z(\Delta z-\partial_{t}z)G_{\lambda}\,dx. \end{aligned}$$

It follows from (2.6) that for each $t \in(0,T)$

2.14 $$\begin{aligned} D_{\lambda}(t) =& \int_{\Omega}\bigl(\vert \nabla y\vert ^{2}+\vert \nabla z\vert ^{2}\bigr)G_{ \lambda}\,dx \\ =& \int_{\Omega}\nabla y\nabla yG_{\lambda}\,dx+ \int_{\Omega}\nabla z\nabla zG_{\lambda}\,dx \\ =& \int_{\Omega}\operatorname{div}(y\nabla yG_{\lambda})\,dx- \int_{\Omega}y \operatorname{div}(\nabla yG_{\lambda})\,dx+ \int_{\Omega}\operatorname{div}(z \nabla zG_{\lambda})\,dx \\ &{}- \int_{\Omega}z\operatorname{div}(\nabla zG_{\lambda })\,dx \\ =&- \int_{\Omega}y\Delta yG_{\lambda}\,dx+ \int_{\Omega}y\nabla y\frac{x-x _{0}}{2(T-t+\lambda)}G_{\lambda}\,dx- \int_{\Omega}z\Delta zG_{\lambda }\,dx \\ &{}+ \int_{\Omega}z\nabla z\frac{x-x_{0}}{2(T-t+\lambda)}G_{\lambda }\,dx \\ =&- \int_{\Omega}y \biggl( \partial_{t}y- \frac{x-x_{0}}{2(T-t+\lambda)} \nabla y+\frac{1}{2}(\triangle y- \partial_{t}y) \biggr) G_{\lambda}\,dx-\frac{1}{2} \int_{\Omega }y(\triangle y-\partial_{t}y)G_{\lambda}\,dx \\ &{}- \int_{\Omega}z \biggl( \partial_{t}z-\frac{x-x_{0}}{2(T-t+\lambda)} \nabla z+\frac{1}{2}(\triangle z-\partial_{t}z) \biggr) G_{\lambda}\,dx \\ &{}- \frac{1}{2} \int_{\Omega}z(\triangle z-\partial_{t}z)G_{\lambda}\,dx. \end{aligned}$$

This, combining with (2.13), shows that

2.15 $$\begin{aligned} 2H'_{\lambda}(t)D_{\lambda}(t) =&- \biggl[2 \int_{\Omega}y \biggl( \partial_{t}y-\frac{x-x _{0}}{2(T-t+\lambda)} \nabla y+\frac{1}{2}(\triangle y-\partial _{t}y) \biggr) G _{\lambda}\,dx \\ &{}+2 \int_{\Omega}z \biggl( \partial_{t}z- \frac{x-x_{0}}{2(T-t+\lambda)} \nabla z+\frac{1}{2}(\triangle z- \partial_{t}z) \biggr) G_{\lambda}\,dx \biggr]^{2} \\ &{}+ \biggl( \int_{\Omega}y(\triangle y-\partial_{t}y)G_{\lambda}\,dx+ \int_{\Omega}z(\triangle z-\partial_{t}z)G_{\lambda}\,dx \biggr) ^{2}. \end{aligned}$$

This completes the proof of this lemma. □

### Lemma 2.4

*For each* $\lambda>0$ *and* $t\in(0,T)$, *then we have*

2.16 $$\begin{aligned} D'_{\lambda}(t) =&- \int_{\partial\Omega} \vert \nabla y\vert ^{2} \partial_{ \nu}G_{\lambda}\,d\sigma+2 \int_{\partial\Omega}\partial_{\nu}y( \nabla y\nabla G_{\lambda})\,d\sigma \\ &{}- \int_{\partial\Omega} \vert \nabla z\vert ^{2}\partial _{\nu}G_{\lambda}\,d\sigma+2 \int_{\partial\Omega}\partial_{\nu}z(\nabla z\nabla G_{ \lambda})\,d\sigma \\ &{}-2 \int_{\Omega} \biggl( \partial_{t}y-\frac{x-x_{0}}{2(T-t+\lambda)} \nabla y+\frac{1}{2}(\triangle y-\partial_{t}y) \biggr) ^{2}G_{\lambda }\,dx \\ &{}-2 \int_{\Omega} \biggl( \partial_{t}z-\frac{x-x_{0}}{2(T-t+\lambda)} \nabla z+\frac{1}{2}(\triangle z-\partial_{t}z) \biggr) ^{2}G_{\lambda }\,dx \\ &{}+\frac{1}{2} \int_{\Omega}(\triangle y-\partial_{t}y)^{2}G_{\lambda }\,dx+ \frac{1}{2} \int_{\Omega}(\triangle z-\partial_{t}z)^{2}G_{\lambda }\,dx +\frac{1}{(T-t+\lambda)}D_{\lambda}(t). \end{aligned}$$

### Proof

By a direct computation, we can derive

2.17 $$\begin{aligned} D'_{\lambda}(t) =&2 \int_{\Omega}\nabla y\partial_{t}\nabla yG_{ \lambda}\,dx+ \int_{\Omega} \vert \nabla y\vert ^{2}\partial _{t}G_{\lambda}\,dx \\ &{}+2 \int_{\Omega}\nabla z\partial_{t}\nabla zG_{\lambda}\,dx+ \int_{\Omega} \vert \nabla z\vert ^{2} \partial_{t}G_{\lambda}\,dx \\ =&2 \int_{\Omega}\nabla y\partial_{t}\nabla yG_{\lambda}\,dx- \int_{\Omega} \vert \nabla y\vert ^{2}\triangle G_{\lambda}\,dx \\ &{}+2 \int_{\Omega}\nabla z\partial_{t}\nabla zG_{\lambda}\,dx- \int_{\Omega} \vert \nabla z\vert ^{2}\triangle G_{\lambda}\,dx \\ =&2 \int_{\Omega}\operatorname{div}(\nabla y\partial_{t}yG_{\lambda})\,dx-2 \int_{\Omega}\partial_{t}y\operatorname{div}(\nabla yG_{\lambda})\,dx- \int_{\Omega} \vert \nabla y\vert ^{2}\triangle G_{\lambda}\,dx \\ &{}+2 \int_{\Omega}\operatorname{div}(\nabla z\partial_{t}zG_{\lambda})\,dx-2 \int_{\Omega}\partial_{t}z\operatorname{div}(\nabla zG_{\lambda})\,dx- \int_{\Omega} \vert \nabla z\vert ^{2}\triangle G_{\lambda}\,dx \\ =&-2 \int_{\Omega}\partial_{t}y\triangle yG_{\lambda}\,dx-2 \int_{ \Omega}\partial_{t}y\nabla y\nabla G_{\lambda}\,dx- \int_{\Omega} \vert \nabla y\vert ^{2}\triangle G_{\lambda}\,dx \\ &{}-2 \int_{\Omega}\partial_{t}z\triangle zG_{\lambda}\,dx-2 \int_{ \Omega}\partial_{t}z\nabla z\nabla G_{\lambda}\,dx- \int_{\Omega} \vert \nabla z\vert ^{2}\triangle G_{\lambda}\,dx \\ =&-2 \int_{\Omega}\partial_{t}y\triangle yG_{\lambda}\,dx+2 \int_{ \Omega}\partial_{t}y\nabla y\frac{x-x_{0}}{2(T-t+\lambda)}G_{\lambda }\,dx- \int_{\Omega} \vert \nabla y\vert ^{2}\triangle G_{\lambda}\,dx \\ &{}-2 \int_{\Omega}\partial_{t}z\triangle zG_{\lambda}\,dx+2 \int_{ \Omega}\partial_{t}z\nabla z\frac{x-x_{0}}{2(T-t+\lambda)}G_{\lambda }\,dx- \int_{\Omega} \vert \nabla z\vert ^{2}\triangle G_{\lambda}\,dx. \end{aligned}$$

However,

2.18 $$\begin{aligned} \vert \nabla y\vert ^{2}\triangle G_{\lambda} =&\operatorname{div}\bigl(\vert \nabla y\vert ^{2} \nabla G_{\lambda}\bigr)-2\operatorname{div} \bigl( \nabla y(\nabla y\nabla G_{ \lambda}) \bigr) \\ &{}+2\triangle y\nabla y\nabla G_{\lambda}+2\sum _{i=1}^{d}\nabla y \partial_{i}y \partial_{i}\nabla G_{\lambda} \end{aligned}$$

and

2.19 $$\begin{aligned} \vert \nabla z\vert ^{2}\triangle G_{\lambda} =&\operatorname{div}\bigl(\vert \nabla z\vert ^{2} \nabla G_{\lambda}\bigr)-2\operatorname{div} \bigl( \nabla z(\nabla z\nabla G_{ \lambda}) \bigr) \\ &{}+2\triangle z\nabla z\nabla G_{\lambda}+2\sum _{i=1}^{d}\nabla z \partial_{i}z \partial_{i}\nabla G_{\lambda}. \end{aligned}$$

Now, we write

2.20 $$\begin{aligned} A = \int_{\partial\Omega} \vert \nabla y\vert ^{2} \partial_{\nu}G_{ \lambda}\,d\sigma-2 \int_{\partial\Omega}\partial_{\nu}y(\nabla y \nabla G_{\lambda})\,d\sigma \end{aligned}$$

and

2.21 $$\begin{aligned} B = \int_{\partial\Omega} \vert \nabla z\vert ^{2} \partial_{\nu}G_{ \lambda}\,d\sigma-2 \int_{\partial\Omega}\partial_{\nu}z(\nabla z \nabla G_{\lambda})\,d\sigma. \end{aligned}$$

Then, by (2.18), (2.19), (2.20), (2.21), we obtain

2.22 $$\begin{aligned} \int_{\Omega} \vert \nabla y\vert ^{2}\triangle G_{\lambda}\,dx =&A +2 \int_{\Omega}\triangle y\nabla y\nabla G_{\lambda}\,dx+2\sum _{i=1} ^{d} \int_{\Omega}\nabla y\partial_{i}y\partial_{i} \nabla G_{\lambda }\,dx \\ =&A -2 \int_{\Omega}\triangle y\nabla y\frac{x-x_{0}}{2(T-t+ \lambda)}G_{\lambda}\,dx \\ &{}+2\sum_{i=1}^{d} \int_{\Omega}\partial_{i}y\partial_{i}y \biggl( \frac {-1}{2(T-t+ \lambda)}G_{\lambda}+\frac{\vert x-x_{0}\vert ^{2}}{4(T-t+\lambda)^{2}}G_{ \lambda} \biggr) \,dx \\ &{}+2\sum_{i\neq j} \int_{\Omega}\partial_{j}y\partial_{i}y \frac{(x _{i}-x_{0i})(x_{j}-x_{0j})}{4(T-t+\lambda)^{2}}G_{\lambda}\,dx \\ =&A -2 \int_{\Omega}\triangle y\nabla y\frac{x-x_{0}}{2(T-t+ \lambda)}G_{\lambda}\,dx+ \int_{\Omega} \vert \nabla y\vert ^{2} \biggl( \frac{-1}{(T-t+ \lambda)} \biggr) G_{\lambda}\,dx \\ &{}+2 \int_{\Omega} \biggl( \frac{x-x_{0}}{2(T-t+\lambda)}\nabla y \biggr) ^{2}G_{\lambda}\,dx. \end{aligned}$$

In the same way, we get

2.23 $$\begin{aligned} \int_{\Omega} \vert \nabla z\vert ^{2}\triangle G_{\lambda}\,dx =&B -2 \int_{\Omega}\triangle z\nabla z\frac{x-x_{0}}{2(T-t+\lambda)}G_{ \lambda}\,dx+ \int_{\Omega} \vert \nabla z\vert ^{2} \biggl( \frac{-1}{(T-t+\lambda )} \biggr) G_{\lambda}\,dx \\ &{}+2 \int_{\Omega} \biggl( \frac{x-x_{0}}{2(T-t+\lambda)}\nabla z \biggr) ^{2}G_{\lambda}\,dx. \end{aligned}$$

Thus, we can rewrite (2.17) as

2.24 $$\begin{aligned} D'_{\lambda}(t) =&-A -B -2 \int_{\Omega} \biggl( \frac{x-x _{0}}{2(T-t+\lambda)}\nabla y \biggr) ^{2}G_{\lambda}\,dx-2 \int_{\Omega } \biggl( \frac{x-x_{0}}{2(T-t+\lambda)}\nabla z \biggr) ^{2}G_{\lambda}\,dx \\ &{}+2 \int_{\Omega}(\triangle y+\partial_{t}y) \frac{x-x_{0}}{2(T-t+ \lambda)}\nabla yG_{\lambda}\,dx+2 \int_{\Omega}(\triangle z+\partial _{t}z) \frac{x-x_{0}}{2(T-t+\lambda)}\nabla zG_{\lambda}\,dx \\ &{}-2 \int_{\Omega}\partial_{t}y\triangle yG_{\lambda}\,dx-2 \int_{ \Omega}\partial_{t}z\triangle zG_{\lambda}\,dx+ \frac{1}{(T-t+\lambda )}D_{\lambda}(t) \\ =&-A -B -2 \int_{\Omega} \biggl( \partial_{t}y-\frac{x-x _{0}}{2(T-t+\lambda)} \nabla y+\frac{1}{2}(\triangle y-\partial _{t}y) \biggr) ^{2}G_{\lambda}\,dx \\ &{}-2 \int_{\Omega} \biggl( \partial_{t}z-\frac{x-x_{0}}{2(T-t+\lambda)} \nabla z+\frac{1}{2}(\triangle z-\partial_{t}z) \biggr) ^{2}G_{\lambda }\,dx \\ &{}+2 \int_{\Omega}\frac{1}{4}(\triangle y+\partial_{t}y)^{2}G_{\lambda }\,dx-2 \int_{\Omega}\partial_{t}y\triangle yG_{\lambda}\,dx \\ &{}+2 \int_{\Omega}\frac{1}{4}(\triangle z+\partial_{t}z)^{2}G_{\lambda }\,dx-2 \int_{\Omega}\partial_{t}z\triangle zG_{\lambda}\,dx+ \frac{1}{(T-t+ \lambda)}D_{\lambda}(t) \\ =&-A -B -2 \int_{\Omega} \biggl( \partial_{t}y-\frac{x-x _{0}}{2(T-t+\lambda)} \nabla y+\frac{1}{2}(\triangle y-\partial _{t}y) \biggr) ^{2}G_{\lambda}\,dx \\ &{}-2 \int_{\Omega} \biggl( \partial_{t}z-\frac{x-x_{0}}{2(T-t+\lambda)} \nabla z+\frac{1}{2}(\triangle z-\partial_{t}z) \biggr) ^{2}G_{\lambda }\,dx \\ &{}+\frac{1}{2} \int_{\Omega}(\triangle y-\partial_{t}y)^{2}G_{\lambda }\,dx+ \frac{1}{2} \int_{\Omega}(\triangle z-\partial_{t}z)^{2}G_{\lambda }\,dx +\frac{1}{(T-t+\lambda)}D_{\lambda}. \end{aligned}$$

This completes the proof of this lemma. □

### Lemma 2.5

*For each* $\lambda>0$ *and* $t\in(0,T)$, *it follows that*

2.25 $$\begin{aligned} \frac{d}{dt}\bigl[(T-t+\lambda)N_{\lambda}(t)\bigr] \leqslant4M^{2}(T+\lambda ). \end{aligned}$$

### Proof

Firstly, we compute $N'_{\lambda}(t)$ as $t\in(0,T)$. By (2.24) and (2.15), we derive that

2.26 $$\begin{aligned} N'_{\lambda}(t) =&2 \biggl( \frac{1}{H_{\lambda}(t)} \biggr) ^{2}\bigl[D'_{ \lambda}(t)H_{\lambda}(t)-D_{\lambda}(t)H'_{\lambda}(t) \bigr] \\ =&\frac{2}{H_{\lambda}(t)} \biggl\{ -A -B +\frac{1}{(T-t+ \lambda)}D_{\lambda}(t) \\ &{}-2 \int_{\Omega} \biggl( \partial_{t}y-\frac{x-x_{0}}{2(T-t+\lambda)} \nabla y+\frac{1}{2}(\triangle y-\partial_{t}y) \biggr) ^{2}G_{\lambda }\,dx+\frac{1}{2} \int_{\Omega}(\triangle y-\partial_{t}y)^{2}G_{\lambda }\,dx \\ &{}-2 \int_{\Omega} \biggl( \partial_{t}z-\frac{x-x_{0}}{2(T-t+\lambda)} \nabla z+\frac{1}{2}(\triangle z-\partial_{t}z) \biggr) ^{2}G_{\lambda }\,dx+\frac{1}{2} \int_{\Omega}(\triangle z-\partial_{t}z)^{2}G_{\lambda }\,dx \biggr\} \\ &{}- \biggl( \frac{1}{H_{\lambda}(t)} \biggr) ^{2} \biggl\{ - \biggl[2 \int_{\Omega}y \biggl( \partial_{t}y-\frac{x-x_{0}}{2(T-t+\lambda)} \nabla y+\frac{1}{2}(\triangle y-\partial_{t}y) \biggr) G_{\lambda}\,dx \\ &{}+2 \int_{\Omega}z \biggl( \partial_{t}z- \frac{x-x_{0}}{2(T-t+\lambda)} \nabla z+\frac{1}{2}(\triangle z- \partial_{t}z) \biggr) G_{\lambda}\,dx \biggr]^{2} \\ &{}+ \biggl[ \int_{\Omega}y(\triangle y-\partial_{t}y)G_{\lambda}\,dx+ \int_{\Omega}z(\triangle z-\partial_{t}z)G_{\lambda}\,dx \biggr] ^{2} \biggr\} \\ =&\frac{1}{(T-t+\lambda)}N_{\lambda}-2\frac{A +B }{H _{\lambda}(t)} \\ &{}+ \biggl( \frac{2}{H_{\lambda}(t)} \biggr) ^{2} \biggl\{ \biggl[ \int_{ \Omega}y \biggl( \partial_{t}y-\frac{x-x_{0}}{2(T-t+\lambda)} \nabla y+ \frac{1}{2}(\triangle y-\partial_{t}y) \biggr) G_{\lambda}\,dx \\ &{}+ \int_{\Omega}z \biggl( \partial_{t}z-\frac{x-x_{0}}{2(T-t+\lambda)} \nabla z+\frac{1}{2}(\triangle z-\partial_{t}z) \biggr) G_{\lambda}\,dx \biggr]^{2} \\ &{}- \int_{\Omega} \biggl( \partial_{t}y-\frac{x-x_{0}}{2(T-t+\lambda)} \nabla y+\frac{1}{2}(\triangle y-\partial_{t}y) \biggr) ^{2}G_{\lambda }\,dx \int_{\Omega}\bigl(y^{2}+z^{2} \bigr)G_{\lambda}\,dx \\ &{}- \int_{\Omega} \biggl( \partial_{t}z-\frac{x-x_{0}}{2(T-t+\lambda)} \nabla z+\frac{1}{2}(\triangle z-\partial_{t}z) \biggr) ^{2}G_{\lambda }\,dx \int_{\Omega}\bigl(y^{2}+z^{2} \bigr)G_{\lambda}\,dx \biggr\} \\ &{}- \biggl( \frac{1}{H_{\lambda}(t)} \biggr) ^{2} \biggl[ \int_{\Omega}y( \triangle y-\partial_{t}y)G_{\lambda}\,dx+ \int_{\Omega}z(\triangle z- \partial_{t}z)G_{\lambda}\,dx \biggr] ^{2} \\ &{}+\frac{1}{H_{\lambda}(t)} \biggl[ \int_{\Omega}(\triangle y-\partial _{t}y)^{2}G_{\lambda}\,dx+ \int_{\Omega}(\triangle z-\partial_{t}z)^{2}G _{\lambda}\,dx \biggr] . \end{aligned}$$

Let

$$\begin{aligned}& \alpha=\partial_{t}y-\frac{x-x_{0}}{2(T-t+\lambda)}\nabla y+ \frac{1}{2}( \triangle y-\partial_{t}y), \\& \beta=\partial_{t}z-\frac{x-x_{0}}{2(T-t+\lambda)}\nabla z+ \frac{1}{2}( \triangle z-\partial_{t}z). \end{aligned}$$

It follows from the Cauchy-Schwarz inequality that

$$\begin{aligned}& \int_{\Omega}\alpha^{2}G_{\lambda}\,dx \int_{\Omega}\bigl(y^{2}+z^{2}\bigr)G _{\lambda}\,dx+ \int_{\Omega}\beta^{2}G_{\lambda}\,dx \int_{\Omega}\bigl(y ^{2}+z^{2} \bigr)G_{\lambda}\,dx \\& \quad = \int_{\Omega}\bigl(\alpha^{2}+\beta^{2} \bigr)G_{\lambda}\,dx \int_{\Omega}\bigl(y ^{2}+z^{2} \bigr)G_{\lambda}\,dx \\& \quad \geq \biggl[ \int_{\Omega}y\alpha G_{\lambda}\,dx+ \int_{\Omega}z \beta G_{\lambda}\,dx \biggr] ^{2}. \end{aligned}$$

It, together with (2.26), shows that

2.27 $$\begin{aligned} \begin{aligned}[b] & N'_{\lambda}(t)-\frac{1}{(T-t+\lambda)}N_{\lambda}(t)+2 \frac{ A +B }{H_{\lambda}(t)} \\ &\quad{} -\frac{1}{H_{\lambda}(t)} \biggl[ \int _{\Omega}(\triangle y-\partial_{t}y)^{2}G_{\lambda}\,dx+ \int_{\Omega }(\triangle z-\partial_{t}z)^{2}G_{\lambda}\,dx \biggr] \leq0. \end{aligned} \end{aligned}$$

In what follows, we will discuss the properties of *A* and *B*. Since $y=z=0$ on *∂*Ω, we have $\nabla y= \partial_{\nu}y\nu$, $\nabla z=\partial_{\nu}z\nu$ on *∂*Ω. If the domain Ω is convex, we can derive that $((x-x_{0})\cdot\nu)\geq0$. According to (2.20) and (2.6), then

$$\begin{aligned} A =& \int_{\partial\Omega} \vert \nabla y\vert ^{2}\partial _{\nu}G_{ \lambda}\,d\sigma-2 \int_{\partial\Omega}\partial_{\nu}y(\nabla y \nabla G_{\lambda})\,d\sigma \\ =&-\frac{1}{2(T-t+\lambda)} \int_{\partial\Omega} \vert \nabla y\vert ^{2}\bigl((x-x _{0})\cdot\nu\bigr)G_{\lambda}\,d\sigma+\frac{1}{(T-t+\lambda)} \\ &{}\times \int_{\partial\Omega}\partial_{\nu}y\bigl((x-x_{0}) \nabla y\bigr)G _{\lambda}\,d\sigma \\ =&-\frac{1}{2(T-t+\lambda)} \int_{\partial\Omega} \vert \nabla y\vert ^{2}\bigl((x-x _{0})\cdot\nu\bigr)G_{\lambda}\,d\sigma+\frac{1}{(T-t+\lambda)} \\ &{}\times \int_{\partial\Omega} \vert \partial_{\nu}y \vert ^{2} \bigl((x-x_{0}) \cdot\nu\bigr)G_{\lambda}\,d\sigma \\ =&\frac{1}{2(T-t+\lambda)}\times \int_{\partial\Omega} \vert \partial _{\nu}y \vert ^{2}\bigl((x-x_{0})\cdot\nu\bigr)G_{\lambda}\,d\sigma \geq0. \end{aligned}$$

In the same way, we get

$$\begin{aligned} B =\frac{1}{2(T-t+\lambda)}\times \int_{\partial\Omega} \vert \partial_{\nu}z \vert ^{2} \bigl((x-x_{0})\cdot\nu\bigr)G_{\lambda }\,d\sigma\geq0. \end{aligned}$$

Combining with (2.27), we get

2.28 $$\begin{aligned} \begin{aligned}[b] & (T-t+\lambda)N'_{\lambda}(t)-N_{\lambda}(t) \\ &\quad{} - \frac{(T-t+\lambda)}{H _{\lambda}(t)} \biggl[ \int_{\Omega}(\triangle y-\partial_{t}y)^{2}G _{\lambda}\,dx+ \int_{\Omega}(\triangle z-\partial_{t}z)^{2}G_{\lambda }\,dx \biggr] \leq0. \end{aligned} \end{aligned}$$

Using equations (1.1) and (2.28) can be written as

$$\begin{aligned} (T-t+\lambda)N'_{\lambda}(t)-N_{\lambda}(t) \leq& \frac{(T-t+ \lambda)}{H_{\lambda}(t)} \biggl[ \int_{\Omega}(ay+bz)^{2}G_{\lambda }\,dx+ \int_{\Omega}(cy+dz)^{2}G_{\lambda}\,dx \biggr] \\ \leq&\frac{4M^{2}(T-t+\lambda)}{H_{\lambda}(t)} \int_{\Omega}\bigl(\vert y\vert ^{2}+\vert z\vert ^{2}\bigr)G _{\lambda}\,dx \\ \leq&4M^{2}(T+\lambda). \end{aligned}$$

Thus,

$$\begin{aligned} \frac{d}{dt} \bigl[ (T-t+\lambda)N_{\lambda}(t) \bigr] \leq4M^{2}(T+ \lambda), \quad \forall t \in(0,T). \end{aligned}$$

This completes the proof of this lemma. □

Let

$$\begin{aligned} \mathcal{K}_{M,y,z,T}=2\ln \biggl( \frac{\int_{\Omega} ( \vert y(x,0)\vert ^{2}+\vert z(x,0)\vert ^{2} ) \,dx}{ \int_{\Omega} ( \vert y(x,T)\vert ^{2}+\vert z(x,T)\vert ^{2} ) \,dx} \biggr) + \frac{m}{T}+8MT+4M^{2}T^{2}+\frac{d}{2}. \end{aligned}$$

Then we have the following lemma.

### Lemma 2.6

*For each* $\lambda>0$, *we have*

2.29 $$\begin{aligned} \lambda N_{\lambda}(T)+\frac{d}{2}\leq \biggl( \frac{\lambda }{T}+1 \biggr) \mathcal{K}_{M,y,z,T}. \end{aligned}$$

### Proof

Integrating (2.25) over $(t,T)$, we have

$$\begin{aligned} \lambda N_{\lambda}(T) \leq&(T-t+\lambda)N_{\lambda}(t)+4M^{2}(T+ \lambda) (T-t) \\ \leq&(T+\lambda)N_{\lambda}(t)+4M^{2}T(T+\lambda). \end{aligned}$$

Integrating the above over $(0,\frac{T}{2})$, we obtain

2.30 $$\begin{aligned} \frac{T}{2}\lambda N_{\lambda}(T)\leq(T+\lambda) \int_{0}^{ \frac{T}{2}}N_{\lambda}(t)\,dt+2M^{2}T^{2}(T+ \lambda). \end{aligned}$$

By integrating (2.11) over $(0,\frac{T}{2})$, we get

$$\begin{aligned} \int_{0}^{\frac{T}{2}}N_{\lambda}(t)\,dt =&- \int_{0}^{\frac{T}{2}}\frac{H'_{ \lambda}(t)}{H_{\lambda}(t)}\,dt+ \int_{0}^{\frac{T}{2}}\frac{2}{H_{ \lambda}(t)} \int_{\Omega} \bigl[ y(\partial_{t}y-\triangle y)+z( \partial_{t}z-\triangle z) \bigr] G_{\lambda}\,dx \,dt \\ =&-\ln\frac{H_{\lambda}(\frac{T}{2})}{H_{\lambda}(0)}+ \int_{0} ^{\frac{T}{2}}\frac{2}{H_{\lambda}(t)} \int_{\Omega} \bigl[ -y(ay+bz)-z(cy+dz) \bigr] G _{\lambda}\,dx \,dt \\ \leq&\ln\frac{H_{\lambda}(0)}{H_{\lambda}(\frac{T}{2})}+ \int_{0} ^{\frac{T}{2}}\frac{2}{H_{\lambda}(t)} \int_{\Omega}2M\bigl(y^{2}+z^{2}\bigr)G _{\lambda}\,dx \,dt \\ \leq&\ln\frac{H_{\lambda}(0)}{H_{\lambda}(\frac{T}{2})}+2MT. \end{aligned}$$

This, alone with (2.30), shows that

$$\begin{aligned} \frac{T}{2}\lambda N_{\lambda}(T)\leq(T+\lambda) \biggl[ \ln \frac{H _{\lambda}(0)}{H_{\lambda}(\frac{T}{2})}+2MT+2M^{2}T^{2} \biggr] . \end{aligned}$$

We have

$$\begin{aligned} \frac{H_{\lambda}(0)}{H_{\lambda}(\frac{T}{2})} =&\frac{\int_{ \Omega} ( \vert y(x,0)\vert ^{2}+\vert z(x,0)\vert ^{2} ) (T+\lambda)^{- \frac{d}{2}}\cdot e^{-\frac{\vert x-x_{0}\vert ^{2}}{4(T+\lambda)}}\,dx}{ \int_{\Omega} ( \vert y(x,\frac{T}{2})\vert ^{2}+\vert z(x,\frac{T}{2})\vert ^{2} ) ( \frac{T}{2}+\lambda)^{-\frac{d}{2}}\cdot e^{-\frac{\vert x-x_{0}\vert ^{2}}{4( \frac{T}{2}+\lambda)}}\,dx} \\ \leq&\frac{\int_{\Omega} ( \vert y(x,0)\vert ^{2}+\vert z(x,0)\vert ^{2} ) \,dx \cdot(\frac{T}{2}+\lambda)^{\frac{d}{2}}}{\int_{\Omega} (\vert y(x, \frac{T}{2})\vert ^{2}+\vert z(x,\frac{T}{2})\vert ^{2} ) \cdot e^{-\frac{\vert x-x _{0}\vert ^{2}}{4(\frac{T}{2}+\lambda)}}\,dx\cdot(T+\lambda )^{\frac{d}{2}}} \\ \leq&\frac{\int_{\Omega} ( \vert y(x,0)\vert ^{2}+\vert z(x,0)\vert ^{2} ) \,dx \cdot(\frac{T}{2}+\lambda)^{\frac{d}{2}}}{\int_{\Omega} (\vert y(x, \frac{T}{2})\vert ^{2}+\vert z(x,\frac{T}{2})\vert ^{2} ) \,dx\cdot(T+\lambda)^{ \frac{d}{2}}}\cdot e^{\frac{m}{4(\frac{T}{2}+\lambda)}} \\ \leq&e^{\frac{m}{2T}}\frac{\int_{\Omega} ( \vert y(x,0)\vert ^{2}+\vert z(x,0)\vert ^{2} ) \,dx}{ \int_{\Omega} ( \vert y(x,\frac{T}{2})\vert ^{2}+\vert z(x,\frac{T}{2})\vert ^{2} ) \,dx}. \end{aligned}$$

Therefore,

$$\begin{aligned} \frac{T}{2}\lambda N_{\lambda}(T) \leq&(T+\lambda) \biggl[ \frac{m}{2T}+ \ln \biggl( \frac{\int_{\Omega} ( \vert y(x,0)\vert ^{2}+\vert z(x,0)\vert ^{2} ) \,dx}{ \int_{\Omega} ( \vert y(x,\frac{T}{2})\vert ^{2}+\vert z(x,\frac{T}{2})\vert ^{2} ) \,dx} \biggr) +2MT+2M ^{2}T^{2} \biggr] . \end{aligned}$$

Using the energy estimate

$$\begin{aligned} \frac{\int_{\Omega} ( \vert y(x,T)\vert ^{2}+\vert z(x,T)\vert ^{2} ) \,dx}{ \int_{\Omega} ( \vert y(x,\frac{T}{2})\vert ^{2}+\vert z(x,\frac{T}{2})\vert ^{2} ) \,dx} \leq e^{2MT}, \end{aligned}$$

we obtain

$$\begin{aligned} \lambda N_{\lambda}(T) \leq& \biggl( \frac{\lambda}{T}+1 \biggr) \biggl[ 2 \ln \biggl( \frac{\int_{\Omega} ( \vert y(x,0)\vert ^{2}+\vert z(x,0)\vert ^{2} ) \,dx}{ \int_{\Omega} ( \vert y(x,T)\vert ^{2}+\vert z(x,T)\vert ^{2} ) \,dx} \biggr) + \frac{m}{T}+8MT+4M^{2}T^{2} \biggr] \\ =& \biggl( \frac{\lambda}{T}+1 \biggr) \biggl( \mathcal{K}_{M,y,z,T}- \frac{d}{2} \biggr) . \end{aligned}$$

Thus,

$$\begin{aligned} \lambda N_{\lambda}(T)+\frac{d}{2} \leq& \biggl( \frac{\lambda}{T}+1 \biggr) \mathcal{K}_{M,y,z,T}-\frac{\lambda}{T}\cdot \frac{d}{2} \\ \leq& \biggl( \frac{\lambda}{T}+1 \biggr) \mathcal{K}_{M,y,z,T}. \end{aligned}$$

This is (2.29). □

While most of the proof of the following lemma is similar to Lemma 3 of [2], we would rather give the proof in detail for the sake of completeness.

### Lemma 2.7

*For each* $T>0$ *and* $y_{0} \in L^{2}(\Omega)$, $z_{0} \in L^{2}( \Omega)$, *the solution* *y* *and* *z*, *with* $y(\cdot,0)=y_{0}(\cdot)$, $z(\cdot,0)=z_{0}(\cdot)$, *to* (1.1) *holds the estimate*:

$$\begin{aligned}& \biggl[ 1-\frac{8\lambda}{r^{2}} \biggl( \frac{\lambda}{T}+1 \biggr) \mathcal{K}_{M,y,z,T} \biggr] \int_{\Omega} \vert x-x_{0}\vert ^{2} \bigl( \bigl\vert y(x,T)\bigr\vert ^{2}+\bigl\vert z(x,T)\bigr\vert ^{2} \bigr) e^{-\frac{\vert x-x_{0}\vert ^{2}}{4\lambda }}\,dx \\& \quad \leq8\lambda \biggl( \frac{\lambda}{T}+1 \biggr) \mathcal{K}_{M,y,z,T} \int_{B_{r}} \bigl( \bigl\vert y(x,T)\bigr\vert ^{2}+\bigl\vert z(x,T)\bigr\vert ^{2} \bigr) e ^{-\frac{\vert x-x_{0}\vert ^{2}}{4\lambda}}\,dx. \end{aligned}$$

### Proof

We first point out the following fact: for any $f \in H_{0}^{1}( \Omega)$ and for each $\lambda>0$, we have $0\leq\int_{\Omega} \vert \nabla ( f(x)\exp(-\frac{\vert x-x_{0}\vert ^{2}}{ 8\lambda}) ) \vert ^{2}\,dx$. By computing the right-hand term, we get

2.31 $$\begin{aligned} \int_{\Omega}\frac{\vert x-x_{0}\vert ^{2}}{8\lambda}\bigl\vert f(x)\bigr\vert ^{2}e^{-\frac{\vert x-x _{0}\vert ^{2}}{4\lambda}}\,dx \leq&2\lambda \int_{\Omega}\bigl\vert \nabla f(x)\bigr\vert ^{2}e ^{-\frac{\vert x-x_{0}\vert ^{2}}{4\lambda}}\,dx \\ &{}+\frac{d}{2} \int_{\Omega}\bigl\vert f(x)\bigr\vert ^{2}e^{-\frac {\vert x-x_{0}\vert ^{2}}{4 \lambda}}\,dx. \end{aligned}$$

It follows from (2.31) and (2.4) that

2.32 $$\begin{aligned}& \int_{\Omega} \vert x-x_{0}\vert ^{2} \bigl( \bigl\vert y(x,T)\bigr\vert ^{2}+\bigl\vert z(x,T)\bigr\vert ^{2} \bigr) e^{-\frac{\vert x-x_{0}\vert ^{2}}{4\lambda}}\,dx \\& \quad \leq 8\lambda \biggl(2\lambda \int_{\Omega} \bigl( \bigl\vert \nabla y(x,T)\bigr\vert ^{2}+\bigl\vert \nabla z(x,T)\bigr\vert ^{2} \bigr) e^{-\frac{\vert x-x_{0}\vert ^{2}}{4\lambda}}\,dx \\& \quad\quad{} +\frac{d}{2} \int_{\Omega} \bigl( \bigl\vert y(x,T)\bigr\vert ^{2}+\bigl\vert z(x,T)\bigr\vert ^{2} \bigr) e ^{-\frac{\vert x-x_{0}\vert ^{2}}{4\lambda}}\,dx \biggr) \\& \quad \leq 8\lambda \biggl( \lambda N_{\lambda}(T)+\frac{d}{2} \biggr) \int_{\Omega} \bigl( \bigl\vert y(x,T)\bigr\vert ^{2}+\bigl\vert z(x,T)\bigr\vert ^{2} \bigr) e^{-\frac{\vert x-x _{0}\vert ^{2}}{4\lambda}}\,dx \\& \quad \leq 8\lambda \biggl( \lambda N_{\lambda}(T)+\frac{d}{2} \biggr) \biggl[ \int_{B_{r}} \bigl( \bigl\vert y(x,T)\bigr\vert ^{2}+\bigl\vert z(x,T)\bigr\vert ^{2} \bigr) e^{-\frac{\vert x-x _{0}\vert ^{2}}{4\lambda}}\,dx \\& \quad\quad{} +\frac{1}{r^{2}} \int_{\Omega\setminus B_{r}}\vert x-x_{0}\vert ^{2} \bigl( \bigl\vert y(x,T)\bigr\vert ^{2}+\bigl\vert z(x,T)\bigr\vert ^{2} \bigr) e ^{-\frac{\vert x-x_{0}\vert ^{2}}{4\lambda}}\,dx \biggr]. \end{aligned}$$

It, combining with (2.29), shows that

2.33 $$\begin{aligned}& \int_{\Omega} \vert x-x_{0}\vert ^{2} \bigl( \bigl\vert y(x,T)\bigr\vert ^{2}+\bigl\vert z(x,T)\bigr\vert ^{2} \bigr) e^{-\frac{\vert x-x_{0}\vert ^{2}}{4\lambda}}\,dx \\& \quad \leq 8\lambda \biggl( \frac{\lambda}{T}+1 \biggr) \mathcal{K}_{M,y,z,T} \biggl[ \int_{B_{r}} \bigl( \bigl\vert y(x,T)\bigr\vert ^{2}+\bigl\vert z(x,T)\bigr\vert ^{2} \bigr) e^{-\frac{\vert x-x _{0}\vert ^{2}}{4\lambda}}\,dx \\& \quad\quad{} +\frac{1}{r^{2}} \int_{\Omega} \vert x-x_{0}\vert ^{2} \bigl( \bigl\vert y(x,T)\bigr\vert ^{2}+\bigl\vert z(x,T)\bigr\vert ^{2} \bigr) e ^{-\frac{\vert x-x_{0}\vert ^{2}}{4\lambda}}\,dx \biggr]. \end{aligned}$$

We get

2.34 $$\begin{aligned}& \biggl[ 1-\frac{8\lambda}{r^{2}} \biggl( \frac{\lambda}{T}+1 \biggr) \mathcal{K}_{M,y,z,T} \biggr] \int_{\Omega} \vert x-x_{0}\vert ^{2} \bigl( \bigl\vert y(x,T)\bigr\vert ^{2}+\bigl\vert z(x,T)\bigr\vert ^{2} \bigr) e^{-\frac{\vert x-x_{0}\vert ^{2}}{4\lambda }}\,dx \\& \quad \leq8\lambda \biggl( \frac{\lambda}{T}+1 \biggr) \mathcal{K}_{M,y,z,T} \int_{B_{r}} \bigl( \bigl\vert y(x,T)\bigr\vert ^{2}+\bigl\vert z(x,T)\bigr\vert ^{2} \bigr) e ^{-\frac{\vert x-x_{0}\vert ^{2}}{4\lambda}}\,dx. \end{aligned}$$

This completes the proof of the lemma. □

## Results and discussion

### The proof of Theorem 1.1

#### Proof

We start with proving (1.4). We take $\lambda>0$ in the estimate of Lemma 2.7 to be such that

3.1 $$\begin{aligned} \frac{8\lambda}{r^{2}} \biggl( \frac{\lambda}{T}+1 \biggr) \mathcal{K} _{M,y,z,T}=\frac{1}{2}. \end{aligned}$$

By direct computation, we have

$$\begin{aligned} \lambda=\frac{1}{2} \biggl( -T+\sqrt{T^{2}+\frac{Tr^{2}}{4\mathcal{K} _{M,y,z,T}}} \biggr) . \end{aligned}$$

Since $\frac{m}{T}\leq\mathcal{K}_{M,y,z,T}$, it follows that

$$\begin{aligned} \frac{1}{\lambda} =&2\frac{T+\sqrt{T^{2}+\frac{Tr^{2}}{4 \mathcal{K}_{M,y,z,T}}}}{\frac{Tr^{2}}{4\mathcal{K}_{M,y,z,T}}} \\ =&8 \biggl( T+\sqrt{T^{2}+\frac{Tr^{2}}{4\mathcal{K}_{M,y,z,T}}} \biggr) \frac{1}{Tr ^{2}}\mathcal{K}_{M,y,z,T} \\ \leq&8 \biggl( 2T+\sqrt{\frac{Tr^{2}}{4\mathcal{K}_{M,y,z,T}}} \biggr) \frac{1}{Tr ^{2}} \mathcal{K}_{M,y,z,T} \\ \leq& \biggl( 16+\frac{4r}{\sqrt{m}} \biggr) \frac{1}{r^{2}} \mathcal{K}_{M,y,z,T} . \end{aligned}$$

Therefore, we have

3.2 $$\begin{aligned} e^{\frac{m}{4\lambda}} \leq&e^{(4m+r\sqrt{m})\frac{1}{r^{2}} \mathcal{K}_{M,y,z,T}} \\ \leq&e^{(4m+r\sqrt{m})\frac{1}{r^{2}}\frac{d}{2}}e^{(4m+r \sqrt{m})\frac{1}{r^{2}}(\frac{m}{T}+8MT+4M^{2}T^{2})} \\ &{}\times \biggl( \frac{\int_{\Omega} ( \vert y(x,0)\vert ^{2}+\vert z(x,0)\vert ^{2} ) \,dx}{ \int_{\Omega} ( \vert y(x,T)\vert ^{2}+\vert z(x,T)\vert ^{2} ) \,dx} \biggr) ^{2(4m+r \sqrt{m})/ r^{2}}. \end{aligned}$$

By Lemma 2.7, we get

$$\begin{aligned} &\int_{\Omega} \vert x-x_{0}\vert ^{2} \bigl( \bigl\vert y(x,T)\bigr\vert ^{2}+\bigl\vert z(x,T)\bigr\vert ^{2} \bigr) e^{-\frac{\vert x-x_{0}\vert ^{2}}{4\lambda}}\,dx \\ &\quad \leq r^{2} \int_{B _{r}} \bigl( \bigl\vert y(x,T)\bigr\vert ^{2}+\bigl\vert z(x,T)\bigr\vert ^{2} \bigr) e ^{-\frac{\vert x-x_{0}\vert ^{2}}{4\lambda}}\,dx. \end{aligned} $$

Then we derive that

$$\begin{aligned}& \int_{\Omega} \bigl( \bigl\vert y(x,T)\bigr\vert ^{2}+\bigl\vert z(x,T)\bigr\vert ^{2} \bigr) e ^{-\frac{m}{4\lambda}}\,dx \\& \quad \leq \int_{\Omega} \bigl( \bigl\vert y(x,T)\bigr\vert ^{2}+\bigl\vert z(x,T)\bigr\vert ^{2} \bigr) e^{-\frac{\vert x-x_{0}\vert ^{2}}{4\lambda}}\,dx \\& \quad \leq \int_{\Omega\cap{ \vert x-x_{0}\vert \geq r}} \bigl( \bigl\vert y(x,T)\bigr\vert ^{2}+\bigl\vert z(x,T)\bigr\vert ^{2} \bigr) e^{-\frac{\vert x-x_{0}\vert ^{2}}{4\lambda }}\,dx \\& \quad \quad{} + \int_{B_{r}} \bigl( \bigl\vert y(x,T)\bigr\vert ^{2}+\bigl\vert z(x,T)\bigr\vert ^{2} \bigr) e ^{-\frac{\vert x-x_{0}\vert ^{2}}{4\lambda}}\,dx \\& \quad \leq \frac{1}{r^{2}} \int_{\Omega} \vert x-x_{0}\vert ^{2} \bigl( \bigl\vert y(x,T)\bigr\vert ^{2}+\bigl\vert z(x,T)\bigr\vert ^{2} \bigr) e^{-\frac{\vert x-x_{0}\vert ^{2}}{4\lambda }}\,dx \\& \quad \quad{} + \int_{B_{r}} \bigl( \bigl\vert y(x,T)\bigr\vert ^{2}+\bigl\vert z(x,T)\bigr\vert ^{2} \bigr) e ^{-\frac{\vert x-x_{0}\vert ^{2}}{4\lambda}}\,dx \\& \quad \leq 2 \int_{B_{r}} \bigl( \bigl\vert y(x,T)\bigr\vert ^{2}+\bigl\vert z(x,T)\bigr\vert ^{2} \bigr) e^{-\frac{\vert x-x_{0}\vert ^{2}}{4\lambda}}\,dx \\& \quad \leq 2 \int_{B_{r}} \bigl( \bigl\vert y(x,T)\bigr\vert ^{2}+\bigl\vert z(x,T)\bigr\vert ^{2} \bigr) \,dx. \end{aligned}$$

Thus,

3.3 $$\begin{aligned}& \int_{\Omega} \bigl( \bigl\vert y(x,T)\bigr\vert ^{2}+\bigl\vert z(x,T)\bigr\vert ^{2} \bigr) \,dx \\& \quad \leq 2e^{\frac{m}{4\lambda}} \int_{B_{r}} \bigl( \bigl\vert y(x,T)\bigr\vert ^{2}+\bigl\vert z(x,T)\bigr\vert ^{2} \bigr) \,dx \\& \quad \leq 2e^{(4m+r\sqrt{m})\frac{1}{r^{2}}\frac{d}{2}}e^{(4m+r \sqrt{m})\frac{1}{r^{2}}(\frac{m}{T}+8MT+4M^{2}T^{2})} \\& \quad \quad{} \times \biggl( \frac{\int_{\Omega} ( \vert y(x,0)\vert ^{2}+\vert z(x,0)\vert ^{2} ) \,dx}{ \int_{\Omega} ( \vert y(x,T)\vert ^{2}+\vert z(x,T)\vert ^{2} ) \,dx} \biggr) ^{2(4m+r \sqrt{m})/ r^{2}} \\& \quad \quad{} \times \int_{B_{r}} \bigl( \bigl\vert y(x,T)\bigr\vert ^{2}+\bigl\vert z(x,T)\bigr\vert ^{2} \bigr) \,dx. \end{aligned}$$

Then we have

$$\begin{aligned}& \int_{\Omega} \bigl( \bigl\vert y(x,T)\bigr\vert ^{2}+\bigl\vert z(x,T)\bigr\vert ^{2} \bigr) \,dx \\& \quad \leq Ce^{\frac{C}{r^{2}}}e^{\frac{C}{Tr^{2}}}e^{\frac{C}{r^{2}}(MT+M ^{2}T^{2})} \biggl( \frac{\int_{\Omega} ( \vert y(x,0)\vert ^{2} +\vert z(x,0)\vert ^{2} ) \,dx}{ \int_{\Omega} ( \vert y(x,T)\vert ^{2}+\vert z(x,T)\vert ^{2} ) \,dx} \biggr) ^{C/ r ^{2}} \\& \quad\quad {}\times \int_{B_{r}} \bigl( \bigl\vert y(x,T)\bigr\vert ^{2}+\bigl\vert z(x,T)\bigr\vert ^{2} \bigr) \,dx, \end{aligned}$$

which is equivalent to the following inequality:

$$\begin{aligned}& \int_{\Omega} \bigl( \bigl\vert y(x,T)\bigr\vert ^{2}+\bigl\vert z(x,T)\bigr\vert ^{2} \bigr) \,dx \\& \quad \leq Ce^{C/T}e^{C(MT+M^{2}T^{2})} \biggl( \int_{\Omega} \bigl( \bigl\vert y(x,0)\bigr\vert ^{2}+\bigl\vert z(x,0)\bigr\vert ^{2} \bigr) \,dx \biggr) ^{\frac{C}{r^{2}+C}} \\& \quad \quad {}\times \biggl( \int_{B_{r}} \bigl( \bigl\vert y(x,T)\bigr\vert ^{2}+\bigl\vert z(x,T)\bigr\vert ^{2} \bigr) \,dx \biggr) ^{\frac{r^{2}}{r^{2}+C}} \\& \quad \leq C\exp \biggl( \frac{C}{T}+C\bigl(MT+M^{2}T^{2} \bigr) \biggr) \biggl( \int_{ \Omega} \bigl( \bigl\vert y_{0}(x)\bigr\vert ^{2}+\bigl\vert z_{0}(x)\bigr\vert ^{2} \bigr) \,dx \biggr) ^{\frac{C}{r ^{2}+C}} \\& \quad \quad {}\times \biggl( \int_{\omega} \bigl( \bigl\vert y(x,T)\bigr\vert ^{2}+\bigl\vert z(x,T)\bigr\vert ^{2} \bigr) \,dx \biggr) ^{\frac{r^{2}}{r^{2}+C}}. \end{aligned}$$

Let $p=\frac{r^{2}}{r^{2}+C}$ and then the above inequality can be written as

3.4 $$\begin{aligned}& \int_{\Omega} \bigl( \bigl\vert y(x,T)\bigr\vert ^{2}+\bigl\vert z(x,T)\bigr\vert ^{2} \bigr) \,dx \\& \quad \leq C \exp \biggl( \frac{C}{T}+C\bigl(MT+M^{2}T^{2}\bigr) \biggr) \biggl( \int_{ \Omega} \bigl( \bigl\vert y_{0}(x)\bigr\vert ^{2}+\bigl\vert z_{0}(x)\bigr\vert ^{2} \bigr) \,dx \biggr) ^{1-p} \\& \quad\quad {}\times \biggl( \int_{\omega} \bigl( \bigl\vert y(x,T)\bigr\vert ^{2}+\bigl\vert z(x,T)\bigr\vert ^{2} \bigr) \,dx \biggr) ^{p}. \end{aligned}$$

Thus, we can obtain (1.4). This completes the proof of this theorem. □

### The proof of Theorem 1.2

#### Proof

In order to give (1.5), we should first prove the following backward uniqueness estimate:

3.5 $$\begin{aligned} \frac{\Vert y(x,0)\Vert _{L^{2}}^{2}+\Vert z(x,0)\Vert _{L^{2}}^{2}}{\Vert y(x,T)\Vert _{L ^{2}}^{2}+\Vert z(x,T)\Vert _{L^{2}}^{2}}\leq\exp \biggl( C(1+2M)Te^{CM^{2}T} \frac{ \Vert y(x,0)\Vert _{H_{0}^{1}}^{2}+\Vert z(x,0)\Vert _{H_{0}^{1}}^{2}}{\Vert y(x,0)\Vert _{L ^{2}}^{2}+\Vert z(x,0)\Vert _{L^{2}}^{2}} \biggr) . \end{aligned}$$

For $y_{0},z_{0}\in H_{0}^{1}(\Omega)$, the solutions of equation (1.1) $y(x,t), z(x,t)\in L^{2}(0,T; H^{2}(\Omega)\cap H_{0} ^{1}(\Omega))$. Then we can define a function $\Phi(t)$ as follows:

$$\begin{aligned} \Phi(t)=\frac{\Vert y(x,t)\Vert _{H_{0}^{1}}^{2}+\Vert z(x,t)\Vert _{H_{0}^{1}}^{2}}{ \Vert y(x,t)\Vert _{L^{2}}^{2}+\Vert z(x,t)\Vert _{L^{2}}^{2}}\geq C. \end{aligned}$$

Let $f=ay+bz$, and $g=cy+dz$. By direct computation, we obtain

3.6 $$\begin{aligned}& \frac{d}{dt} \biggl( \frac{1}{2}\Vert y\Vert _{L^{2}}^{2} \biggr) =-\Vert y\Vert _{H_{0} ^{1}}^{2}- \langle ay+bz,y\rangle, \end{aligned}$$

3.7 $$\begin{aligned}& \frac{d}{dt} \biggl( \frac{1}{2}\Vert z\Vert _{L^{2}}^{2} \biggr) =-\Vert z\Vert _{H_{0} ^{1}}^{2}- \langle cy+dz,z\rangle, \\& \frac{d}{dt} \biggl( \frac{1}{2}\Vert y\Vert _{H_{0}^{1}}^{2} \biggr) =-\Vert \triangle y\Vert _{L^{2}}^{2}+ \int_{\Omega}\triangle y(ay+bz)\,dx, \\& \frac{d}{dt} \biggl( \frac{1}{2}\Vert z\Vert _{H_{0}^{1}}^{2} \biggr) =-\Vert \triangle z\Vert _{L^{2}}^{2}+ \int_{\Omega}\triangle z(cy+dz)\,dx. \end{aligned}$$

Then

$$\begin{aligned} \frac{d}{dt}\Phi(t) =&\frac{ ( \Vert y\Vert _{H_{0}^{1}}^{2}+\Vert z\Vert _{H_{0} ^{1}}^{2} ) ' ( \Vert y\Vert _{L^{2}}^{2}+\Vert z\Vert _{L^{2}}^{2} ) - ( \Vert y\Vert _{H_{0}^{1}}^{2}+\Vert z\Vert _{H_{0}^{1}}^{2} ) ( \Vert y\Vert _{L^{2}}^{2}+\Vert z\Vert _{L^{2}}^{2} ) '}{ ( \Vert y\Vert _{L^{2}}^{2}+\Vert z\Vert _{L^{2}}^{2} ) ^{2}} \\ =&\frac{2}{ ( \Vert y\Vert _{L^{2}}^{2}+\Vert z\Vert _{L^{2}}^{2} ) ^{2}} \\ &{}\times\biggl\{ \biggl( -\Vert \triangle y\Vert _{L^{2}}^{2}-\Vert \triangle z\Vert _{L^{2}} ^{2}+ \int_{\Omega}f\triangle y\,dx+ \int_{\Omega}g\triangle z\,dx \biggr) \bigl( \Vert y\Vert _{L^{2}}^{2}+\Vert z\Vert _{L^{2}}^{2} \bigr) \\ &{}+ \biggl( \Vert y\Vert _{H_{0}^{1}}^{2}+\Vert z\Vert _{H_{0}^{1}}^{2}+ \int_{\Omega}fy\,dx+ \int_{\Omega}gz\,dx \biggr) \bigl( \Vert y\Vert _{H_{0}^{1}}^{2}+\Vert z\Vert _{H_{0}^{1}} ^{2} \bigr) \biggr\} \\ =&\frac{2}{ ( \Vert y\Vert _{L^{2}}^{2}+\Vert z\Vert _{L^{2}}^{2} ) ^{2}} \\ &{}\times \biggl\{ \biggl( -\Vert \triangle y\Vert _{L^{2}}^{2}-\Vert \triangle z\Vert _{L^{2}} ^{2}+ \int_{\Omega}f\triangle y\,dx+ \int_{\Omega}g\triangle z\,dx \biggr) \bigl( \Vert y\Vert _{L^{2}}^{2}+\Vert z\Vert _{L^{2}}^{2} \bigr) \\ &{}+ \biggl( \Vert y\Vert _{H_{0}^{1}}^{2}+\Vert z\Vert _{H_{0}^{1}}^{2}+\frac{1}{2} \biggl( \int _{\Omega}fy\,dx+ \int_{\Omega}gz\,dx \biggr) \biggr) ^{2}-\frac{1}{4} \biggl( \int _{\Omega}fy\,dx+ \int_{\Omega}gz\,dx \biggr) ^{2} \biggr\} . \end{aligned}$$

Integrating by parts and using Cauchy-Schwarz inequality, we have

$$\begin{aligned} \frac{d}{dt}\Phi(t) =&\frac{2}{ ( \Vert y\Vert _{L^{2}}^{2}+\Vert z\Vert _{L^{2}} ^{2} ) ^{2}} \\ &{}\times\biggl\{ \biggl( -\Vert \triangle y\Vert _{L^{2}}^{2}-\Vert \triangle z\Vert _{L^{2}}^{2}+ \int_{\Omega}f\triangle y\,dx+ \int_{\Omega }g\triangle z\,dx \biggr) \bigl( \Vert y\Vert _{L^{2}}^{2}+\Vert z\Vert _{L^{2}}^{2} \bigr) \\ &{}+ \biggl( \int_{\Omega}\biggl(-\triangle y+\frac{f}{2}\biggr)y\,dx+ \int_{\Omega}\biggl(- \triangle z+\frac{g}{2}\biggr)z\,dx \biggr) ^{2}-\frac{1}{4} \biggl( \int_{\Omega }fy\,dx+ \int_{\Omega}gz\,dx \biggr) ^{2} \biggr\} \\ \leq&\frac{2}{ ( \Vert y\Vert _{L^{2}}^{2}+\Vert z\Vert _{L^{2}}^{2} ) ^{2}} \\ &{}\times \biggl\{ \biggl( -\Vert \triangle y\Vert _{L^{2}}^{2}-\Vert \triangle z\Vert _{L^{2}} ^{2}+ \int_{\Omega}f\triangle y\,dx+ \int_{\Omega}g\triangle z\,dx \biggr) \bigl( \Vert y\Vert _{L^{2}}^{2}+\Vert z\Vert _{L^{2}}^{2} \bigr) \\ &{}+ \biggl( \biggl\Vert -\triangle y+\frac{f}{2}\biggr\Vert _{L^{2}}\Vert y\Vert _{L^{2}}+\biggl\Vert -\triangle z+ \frac{g}{2}\biggr\Vert _{L^{2}}\Vert z\Vert _{L^{2}} \biggr) ^{2}-\frac{1}{4} \biggl( \int_{\Omega}fy\,dx+ \int_{\Omega }gz\,dx \biggr) ^{2} \biggr\} \\ \leq&\frac{2}{ ( \Vert y\Vert _{L^{2}}^{2}+\Vert z\Vert _{L^{2}}^{2} ) ^{2}} \\ &{}\times\biggl\{ \biggl( -\Vert \triangle y\Vert _{L^{2}}^{2}-\Vert \triangle z\Vert _{L^{2}} ^{2}+ \int_{\Omega}f\triangle y\,dx+ \int_{\Omega}g\triangle z\,dx \biggr) \bigl( \Vert y\Vert _{L^{2}}^{2}+\Vert z\Vert _{L^{2}}^{2} \bigr) \\ &{}+ \biggl( \biggl\Vert -\triangle y+\frac{f}{2}\biggr\Vert _{L^{2}}^{2}+\biggl\Vert - \triangle z+\frac{g}{2} \biggr\Vert _{L^{2}}^{2} \biggr) \bigl( \Vert y\Vert _{L^{2}}^{2}+\Vert z\Vert _{L^{2}}^{2} \bigr) \biggr\} . \end{aligned}$$

Thus,

$$\begin{aligned} \frac{d}{dt}\Phi(t) \leq&\frac{2}{ ( \Vert y\Vert _{L^{2}}^{2}+\Vert z\Vert _{L^{2}}^{2} ) ^{2}} \biggl( \biggl\Vert \frac{f}{2}\biggr\Vert _{L^{2}}^{2}+ \biggl\Vert \frac{g}{2}\biggr\Vert _{L^{2}}^{2} \biggr) \bigl( \Vert y\Vert _{L^{2}}^{2}+\Vert z\Vert _{L^{2}}^{2} \bigr) \\ =&\frac{\Vert f\Vert _{L^{2}}^{2}+\Vert g\Vert _{L^{2}}^{2}}{2 ( \Vert y\Vert _{L^{2}}^{2}+\Vert z\Vert _{L^{2}}^{2} ) }=\frac{\Vert ay+bz\Vert _{L ^{2}}^{2}+\Vert cy+dz\Vert _{L^{2}}^{2}}{2 ( \Vert y\Vert _{L^{2}}^{2}+ \Vert z\Vert _{L^{2}}^{2} ) } \\ \leq& CM^{2}\frac{\Vert y\Vert _{H_{0}^{1}}^{2}+\Vert z\Vert _{H_{0}^{1}}^{2}}{\Vert y \Vert _{L^{2}}^{2}+\Vert z\Vert _{L^{2}}^{2}}=CM^{2}\Phi(t). \end{aligned}$$

With the aid of Gronwall’s inequality, we obtain, for $t \in(0,T)$,

3.8 $$\begin{aligned} \Phi(t)\leq e^{CM^{2}T}\Phi(0). \end{aligned}$$

This, combining with (3.6) and (3.7), indicates that

3.9 $$\begin{aligned} 0 =&\frac{1}{2}\frac{d}{dt} \bigl( \Vert y\Vert _{L^{2}}^{2}+\Vert z\Vert _{L^{2}}^{2} \bigr) + \Vert y\Vert _{H_{0}^{1}}^{2}+\Vert z\Vert _{H_{0}^{1}}^{2}+\langle ay+bz,y\rangle+ \langle cy+dz,z\rangle \\ \leq&\frac{1}{2}\frac{d}{dt} \bigl( \Vert y\Vert _{L^{2}}^{2}+\Vert z\Vert _{L^{2}} ^{2} \bigr) +\Phi(t) \bigl( \Vert y\Vert _{L^{2}}^{2}+\Vert z \Vert _{L^{2}}^{2} \bigr) +2M \Phi(t) \bigl( \Vert y\Vert _{L^{2}}^{2}+\Vert z\Vert _{L^{2}}^{2} \bigr) \\ \leq&\frac{1}{2}\frac{d}{dt} \bigl( \Vert y\Vert _{L^{2}}^{2}+\Vert z\Vert _{L^{2}} ^{2} \bigr) +(1+2M)e^{CM^{2}T}\Phi(0) \bigl( \Vert y\Vert _{L^{2}}^{2}+ \Vert z\Vert _{L^{2}}^{2} \bigr) . \end{aligned}$$

Integrating (3.9) on $(0,T)$, we get the desired estimate

$$\begin{aligned} \bigl\Vert y(x,0)\bigr\Vert _{L^{2}}^{2}+\bigl\Vert z(x,0)\bigr\Vert _{L^{2}}^{2}\leq\exp \bigl( 2(1+2M)Te ^{CM^{2}T}\Phi(0) \bigr) \bigl( \bigl\Vert y(x,T)\bigr\Vert _{L^{2}}^{2}+\bigl\Vert z(x,T)\bigr\Vert _{L ^{2}}^{2} \bigr) . \end{aligned}$$

Thus,

$$\begin{aligned} \frac{\Vert y(x,0)\Vert _{L^{2}}^{2}+\Vert z(x,0)\Vert _{L^{2}}^{2}}{\Vert y(x,T)\Vert _{L ^{2}}^{2}+\Vert z(x,T)\Vert _{L^{2}}^{2}}\leq\exp \biggl( 2(1+2M)Te^{CM^{2}T}\frac{ \Vert y(x,0)\Vert _{H_{0}^{1}}^{2}+\Vert z(x,0)\Vert _{H_{0}^{1}}^{2}}{\Vert y(x,0)\Vert _{L ^{2}}^{2}+\Vert z(x,0)\Vert _{L^{2}}^{2}} \biggr) . \end{aligned}$$

This is the proof of (3.5), and we have completed the proof of Theorem 1.2. □

## Conclusions

In this work, we discuss the unique continuation for the linear coupled heat equations. Our results demonstrate that the value of the solution equation (1.1) can be determined uniquely by its value on an arbitrary open subset *ω* of Ω at any given positive time *T*. In other words, if the solution of equation (1.1) satisfies $y(\cdot,T)=z(\cdot,T)=0$ on *ω*, then $y=z=0$ on $\Omega \times(0,T)$. This is the quantitative version of the unique continuation property for equation (1.1).
